# Evidence-based quality criteria in research, development and application of Chinese medicines

**DOI:** 10.1186/1479-5876-10-S2-A37

**Published:** 2012-10-17

**Authors:** Zhengtao Wang

**Affiliations:** 1 Institute of Chinese Materia Medica, Shanghai University of Traditional Chinese Medicine, Shanghai, China

## 

TCM mainly consists of crude drugs, slices for decoction, extracts and Chinese patent medicine preparations. Crude drugs are the raw and starting materials of preparation of slices, extracted intermediates and the finished products.

The crude drugs being collected or cultivated from nature has many factors affecting its quality. Different species/varieties,different produce areas, different harvest times , different processing methods and different storage conditions can all cause an enormous diversity on the quality.

It has been evidenced that the multi-originated herbs showed diverse in chemical profiles and consequently un-equivalent in pharmacological potency. This situation may be well recognized by pharmacogosists, however are often neglect or ignored by pharmacologists and TCM doctors in the biological activity screening, in non-clinical investigation research, and in clinical trials, which undoubtedly led to unrepeatability of the results and even wrong conclusive direction.

As examples, four species of Rheum are recorded for Rhubarb in Chinese Pharmacopoeia but only one or two species possess diarrhea effects. As for Curcumae Radix, only the root of Curcuma longa L showed potent choleretic activity among the fure species of Curcuma recorded in Chinese Pharmacopoeia.

In view of this consideration, the importance of a pre-experiment assessment of the starting material and the standardized and advanced methodologies for the authentication and quality evaluation of TCM herbs/products will be addressed in this presentation.

